# Effect of Luminance and Contrast Variation on Stereoacuity Measurements Using Smartphone Technology

**DOI:** 10.1155/2021/5258782

**Published:** 2021-12-23

**Authors:** Lu Liu, Lingxian Xu, Junyue Wang, Huang Wu

**Affiliations:** ^1^Department of Optometry, Second Hospital of Jilin University, Changchun, China; ^2^Hospital of Stomatology, Jilin University, Changchun, China

## Abstract

Owing to the limitations of printed stereoacuity tests, the effects of luminance and contrast on stereopsis have not yet been sufficiently investigated, despite its important implications in designing stereoacuity measuring instruments, particularly for electronic devices. A stereopsis measurement system was established using two 4 K smartphones and a phoropter to evaluate the effects of luminance and contrast variations on the stereoacuity test. Seventeen young subjects with normal visual acuity and stereopsis were recruited. Two types of test symbols, contour-based and random-dot-based, were used in the experiment. Four series tests were established with different maximum brightness values, including 240 lux, 120 lux, 60 lux, and 30 lux. Each series test contained 19 pages with different contrasts between 95% and 5% and was calculated using the Michelson contrast formula. No significant difference was found for both contour-based and random-dot-based stereograms in any of the contrast groups with different maximum brightness. Similarly, no significant difference was found between contour-based and random-dot-based patterns under different contrasts of above 35%. As the contrast decreased below 30%, the stereopsis was significantly better in the contour-based pattern than in the random-dot-based pattern for some degrees of contrast. The luminance and contrast of the digital display are not critical factors for stereoacuity under normal circumstances. This implies that a standard monitor with a certain 3D technology can be used to measure the stereoacuity threshold without calibrating the luminance and contrast.

## 1. Introduction

Stereopsis is a binocular vision processing system that helps in detecting the distance between objects accurately, and it is a commonly used index for clinically evaluating binocular vision. The luminance and contrast of the test material may affect the threshold of stereopsis under extreme conditions [1–3]. However, the results of a stereopsis test under normal environmental conditions may not be affected. The luminance and contrast should not be changed during the Frisby stereo test, a “real depth” test [4, 5], when conducting an examination. Polaroid spectacles, utilized in Titmus stereo test, etc. [6, 7], would reduce luminance by approximately 40% for both the eyes [8]. Anaglyph spectacles, adopted in TNO stereo test [9–11], affect not only the luminance, but also the contrast between binoculars. Although the test results may not exhibit a high agreement between different types of examinations, the difference should be within a clinically acceptable range.

With the development of computer technology, an increasing number of applications have been used to evaluate visual function with video terminals, such as visual acuity [12], color vision [13], and contrast sensitivity [14]. Moreover, the 3D effect has already been achieved with computer assistance and is extensively used in video games or film making [15–17]. Polarization 3D, 3D liquid crystal shutter glasses, and glasses-free 3D technology have been developed to achieve 3D expression. Some researchers have already used polarization techniques or 3D liquid crystal shutter glasses to detect the threshold of stereoacuity [18–23]. Handheld mobile terminals have been used as effective instruments for evaluating stereopsis. Rodríguez-Vallejo et al. established a stereoacuity test called TST, performed on an iPad application [24]. Bonfanti et al. presented a “stereo acuity test using a smartphone inserted into a Google Cardboard” [25]. We have conducted research on stereopsis using two 4 K smartphones [26–29] and autostereoscopic smartphones [30, 31]. The disparity in computer expression can be precisely determined by the pixel arrangement characteristics of the monitor, but the luminance and contrast of different displays cannot be easily controlled. The effects of variations in luminance and contrast on stereopsis threshold tests conducted with video terminals has not been evaluated in detail. Our study aims to explore variations in the stereoacuity threshold under different symbols and background contrasts, evaluate the tolerance range for an acceptable result, and subsequently judge whether the contrast is an essential influencing factor in normal 3D displays.

## 2. Materials and Methods

### 2.1. Test System

**Figure 1:**
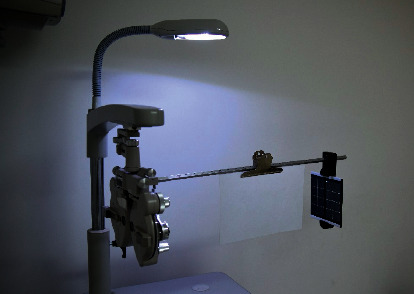
Photograph of the actual test.

A stereopsis measurement system was established with two 4 K smartphones and a phoropter, as previously used [26–29]. The resolution of the smartphone screen was 3840 × 2160 (Sony Xperia XZ Premium; Sony Mobile Communications Inc., Tokyo, Japan). With the aid of two approximately 5.5Δ based-out Risley prisms, two smartphones may create a minimum 10 second of arc (arcsec, ″) disparity at a checking distance of 0.65 m at the near vision test rod of the phoropter (Topcon VT-10, Topcon Corp, Tokyo, Japan) (Figure 1). The disparity could be calculated by using the formula: disparity = [(*n*·*w*)/*d*] (180/*π* × 3600), where *w* is the physical width of one pixel of the smartphone, *n* is the shifting number of pixels, *d* is the viewing distance, 180/*π* converts radians to degrees, and the number 3600 converts degrees to arcsecs.

### 2.2. Test Symbols

In this experiment, the Michelson contrast was adopted. We designed a pattern in which the proportion of the relatively light elements and the relatively dark elements was closer to 50%, to meet the demand of the Michelson contrast.

1. *Contour-Based Symbols.* The background of the pattern was a black and white checkerboard mode, and the size of the square was 108 × 108 pixels. The stereo test unit imitated the quantitative measurements of Stereo Fly test (Stereo Optical Company, Inc. Illinois, USA). The stereo circle could appear at the up, down, left, or right positions randomly. The stereo symbol appeared out of the plane because the disparity setting was all crossed. The target circle could be distinguished when the stereopsis threshold of the subject was better than the setting disparity.
2. *Random-Dot-Based Symbols.* The test symbol was an imitation of Pacman in the TNO stereo test (Lameris Ootech BV, Ede, Netherlands). The “mouth” of the Pacman may face up, down, left, or right direction at random. The subject had to determine the location of the mouth of the Pacman when the stereopsis threshold was better than the setting disparity. The minimal size of the random dot was 6 × 6 pixels (equivalent to 0 logMAR resolution) to ensure that all dots could be distinguished by the participant [32].

### 2.3. Test Pages

#### 2.3.1. Establish a Grayscale Image Library

In this experiment, the Michelson contrast was adopted. The Michelson contrast formula was determined as *C* = (*L*_max_ − *L*_min_)/(*L*_max_ + *L*_min_), where *L*_max_ and *L*_min_ represent the highest and lowest luminance, respectively. The areas of *L*_max_ and *L*_min_ were approximately the same, and the luminance of *L*_max_ and *L*_min_ were the actual brightness. The evaluation tools of the experiment were smartphones, and all test images were created using a computer. The color and grayscale shown on the screen were created using RGB code. RGB stands for red, green, and blue, which have integer values from 0 to 255. They produce the color space shown on the display. When R = G = B, the color is transferred to the gray system utilized in this test. The maximum brightness (code 100%) corresponded to RGB = (255, 255, 255) and the minimum brightness (code 0) corresponded to RGB = (0, 0, 0). Using the RGB code, we drew 101 paintings representing code brightness between 100% and 0%. A screen luminance meter (SM208, M&A Instrument Inc., Shenzhen, China) was used to measure the brightness of each of the 101 images. Therefore, a corresponding relationship could be established between the code grayscale and the real brightness. The maximum luminance of the smartphone used was higher than 280 lux. In practice, we adjusted the maximum brightness to 240 lux. A grayscale image library, including 101 grayscale images from grayscale no. 100 to grayscale no. 0, was established. Grayscale no. 100 represented a code brightness of 100%, an RGB code of (255, 255, 255), and an actual brightness of 240 lux; grayscale no. 99 represented a code brightness of 99%, an RGB code of (252, 252, 252), and an actual brightness of 237 lux; and so on, until grayscale no. 0 (code brightness 0%, RGB value (0,0,0), actual brightness 0.2 lux).

When an image is drawn with two different grayscale codes from the library, the Michelson contrast can be calculated. For example, if *L*_max_ is set to grayscale no. 70 (actual brightness 117 lux) and *L*_min_ is set to grayscale no. 30 (actual brightness 18 lux), then the contrast is 73%. However, if we assign a contrast value and an *L*_max_ value, the *L*_min_ value can also be calculated. For example, if the contrast is set to 40% and *L*_max_ is set to grayscale no. 70 (actual brightness 117 lux), then *L*_min_ would be grayscale no. 48 (actual brightness 50 lux). This means that if an image is drawn using the grayscale no. 70 and grayscale no. 48, the Michelson contrast would be 40%. A total of 101 grayscale images formed the library from which we could choose a suitable image code to create every contrast we needed.

#### 2.3.2. Establishing Four Series Tests Pages

Four series of tests were established with different maximum brightness values. Group 1 had an actual maximum brightness of 240 lux (grayscale no. 100); group 2 of 120 lux (grayscale no. 71); group 3 of 60 lux (grayscale no. 52); and for group 4, the actual maximum brightness was 30 lux (grayscale no. 38). Each series test contained 19 pages with different contrasts between 95% and 5%. For example, if we wanted to create a 95% contrast image in group 1, the minimum brightness would be 6.2 lux, as calculated from the Michelson contrast formula. This corresponds to the grayscale no. 19 in the library. Thus, we used grayscale no. 100 (RGB [255, 255, 255]) and grayscale no. 19 (RGB [48, 48, 48]) for drawing the test image. If we wanted to create a 45% contrast image in group 3, we used the grayscale no. 52 (RGB [133, 133, 133], an actual brightness of 60 lux) and grayscale no. 34 (RGB [87, 87, 87], actual brightness 22.75 lux) to draw the test image. The maximum brightness was kept unchanged in each group, while the minimum brightness was changed according to the contrast we set.

#### 2.3.3. Determination of the Threshold of Stereopsis

**Figure 2:**
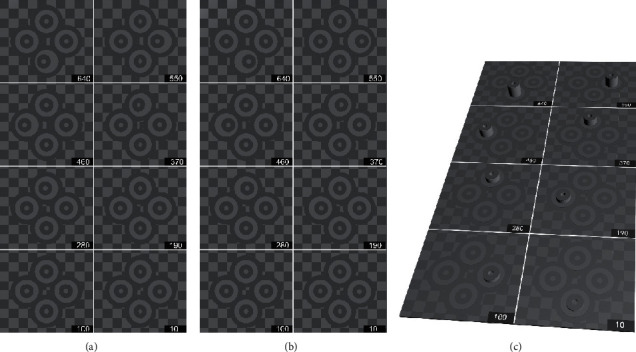
Legend of contour-based pattern of the first-grade test page with 30% contrast for a maximum brightness of 60 lux. (a) Picture viewed by the left eye. (b) Picture viewed by the right eye. (c) Simulation of the perception generated by the test images. The luminance of the dark and light elements was 32 lux (grayscale no. 39, RGB [99, 99, 99]) and 60 lux (grayscale no. 52, RGB [133, 133, 133]), respectively. The setting disparity of the stereo ring was 640″ (down), 550″ (right), 460″ (left), 370″ (up), 280″ (up), 190″ (left), 100″ (right), and 10″ (down). If the stereopsis of a subject is better than the setting disparity, the stereo target may appear out of the plane when fusing correctly.

**Figure 3:**
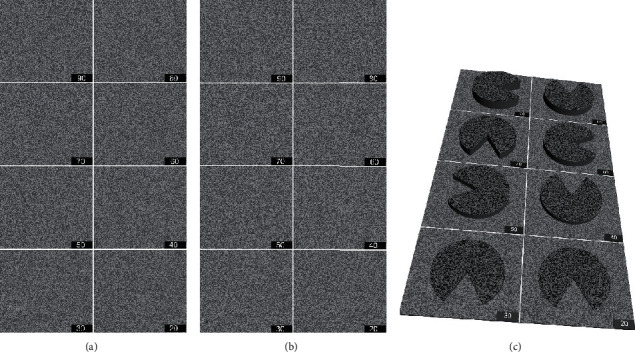
Legend of random-dot-based pattern of the first page of the second-grade test page with 50% contrast for a maximum brightness of 120 lux. (a) Picture viewed by the left eye. (b) Picture viewed by the right eye. (c) Simulation of the perception generated by the test images. The luminance of the dark and light elements was 40 lux (grayscale no. 43, RGB [110, 110, 110]) and 120 lux (grayscale no. 71, RGB [181, 181, 181]), respectively. The setting disparity of the mouth of the Pacman was 90″ (facing right), 80″ (facing up), 70″ (facing down), 60″ (facing right), 50″ (facing left), 40″ (facing up), 30″ (facing down), and 20″ (facing down). If the stereopsis of a subject is better than the setting disparity, the mouth of the Pacman should be distinguished when fusing correctly.

Both the contour-based test and random-dot-based test utilized two grade test patterns. Each test page contained 8 test units of different disparities. The test step range for the first grade was 90″, from 10″ to 640″. The specific parameters were 10″, 100″, 190″, 280″, 370″, 460″, 550″, and 640″. There were seven pages in the second-grade test, including 20″ to 90″, 110″ to 180″, 200″ to 270″, 290″ to 360″, 380″ to 450″, 470″ to 540″, and 560″ to 630″, with step range of 10″ inside each test page (Figures 2 and 3). A program written in C# was used to produce all stereograms.

### 2.4. Test Procedure

**Figure 4:**
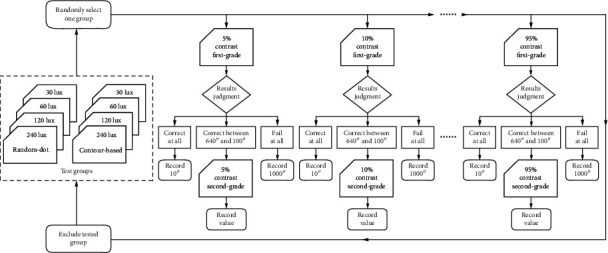
The flowchart of test procedure. Test groups included 4 random-dot-based patterns (maximum brightness 240 lux, 120 lux, 60 lux, or 30 lux) and 4 contour-based patterns (maximum brightness 240 lux, 120 lux, 60 lux, or 30 lux). One group was selected randomly to test a participant. Two-step choices were conducted to measure the stereopsis threshold of 5% contrast, and then 10% contrast, and so on, until 95% contrast. Randomly, another group was chosen to do the examination again, and so on, until finishing all the 8 groups' tests.

The test sequence of the contour-based pattern or random-dot-based pattern was randomly selected. The sequence of the maximum brightness, including 240 lux, 120 lux, 60 lux, or 30 lux, was also randomly selected. However, the test sequence of the contrast ranged from 5% to 95%. At the beginning of the test, a first-grade page with 5% contrast was shown, and the subject was asked to distinguish the stereo target from 640″ to 10″. If the subject could do this correctly at 280″ but failed at 190″, turn to page 3 in grade 2 (including 200″ to 270″). If the subject could choose correctly at 240″ but failed at 230″, then, 240″ was recorded as the stereopsis threshold of the subject. If the subject answered correctly at 10″ in grade 1, the stereopsis value of the subject was recorded as 10″. If the subject failed at 640″, the stereopsis value was recorded as 1000″. The flowchart of test procedure is shown in Figure 4.

### 2.5. Subjects

A total of 17 subjects (5 men and 12 women), aged 20 to 28 years, were recruited. None of the best-corrected visual acuity of each eye was worse than 0 logMAR. The stereoacuity for all participants was no worse than 40″ as evaluated by the Fly Stereo Acuity Test (Vision Assessment Corporation, Illinois, USA). All participants provided informed written consent before participating in the study. The research protocol observed the tenets of the Declaration of Helsinki and was approved by the ethics committee of the Second Hospital of Jilin University (No. 2020-110).

### 2.6. Statistical Analysis

All data were processed using PASW Statistic 18.0 (IBM SPSS Inc.). The Shapiro–Wilk test was used to explore the distribution of the data. If the data satisfied normal distribution patterns, parametric tests were used (one-way ANOVA tests were applied to analyze the differences within those groups and paired *t*-tests were applied to analyze the differences between two groups). If the data were not normally distributed, nonparametric tests were carried out (the Kruskal–Wallis test was used to analyze the differences within those groups, and the Wilcoxon signed-rank test was used to test the difference between two groups).

## 3. Results

### 3.1. Effect of Actual Luminance and Contrast of Screen

None of the data followed a normal distribution (Shapiro–Wilk test, *P* all <0.05). A nonparametric test was used to analyze the data. Four groups with a different maximum luminance of the screen (group 1: 240 lux, group 2: 120 lux, group 3: 60 lux, and group 4: 30 lux) were compared under the same contrast. No significant difference was found in both contour-based and random-dot-based stereograms for the 95% to 5% contrast range (Kruskal–Wallis test, *P* all >0.05; Table 1).

### 3.2. Difference between Random-Dot-Based and Contour-Based Patterns According to Contrast

**Figure 5:**
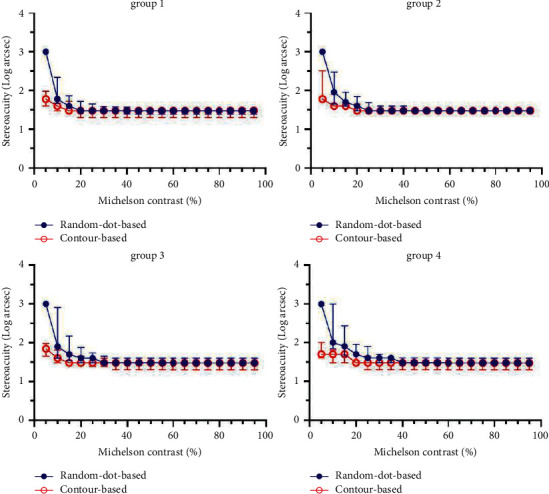
Trend curves characterized by the relationship between stereoacuity and Michelson contrast for different test symbols. Groups 1, 2, 3, and 4 represent the maximum brightness values at 240, 120, 60, and 30 lux, respectively. The circles and bars represent the median and quartile of the stereopsis, respectively.

No significant difference was found between random-dot-based and contour-based patterns under different contrasts as long as the contrast was no lower than 35% (Wilcoxon signed-rank test, *P* all >0.05). When the contrast decreased below 30%, differences appeared in four groups. The stereopsis was significantly better for the contour-based pattern than for the random-dot-based pattern for some degrees of contrast (group 1 & group 2, ≤25%; group 3 & group 4, ≤30%; see Table 2). All participants failed at the 5% contrast test with the random-dot pattern, and none of them failed the contour-based pattern. The relationship between contrast and stereoacuity is shown in Figure 5.

## 4. Discussion

The effect of contrast on stereopsis has been discussed for several decades. Traditional stereopsis measurement tools, such as the Titmus stereoacuity test, TNO, or FD2, have difficulty in manipulating luminance; therefore, it is difficult to perform the experiment with these tools. The computer-aided stereoacuity test is a powerful tool for this purpose.

Li et al. investigated the effects of luminance contrast on the stereoscopic threshold using pairs of random dot stereograms. The stimuli were generated on a system consisting of a monitor and a graphic device driven by an AST 286 computer. They found that stereoacuity could be maintained in the best state until the contrast dropped to 30% [33]. Legge et al. used vertical sine-wave gratings to evaluate stereopsis with different contrasts. A computer (LSI-11/23) with a CRT monitor was used to generate all stereo targets. They found that the stereopsis threshold was inversely proportional to the square root of the contrast [1]. Halpern et al. discovered that stereoacuity varied proportionally with the square of the contrast under low contrast conditions, while the model was independent of contrast at higher contrast conditions [2]. Cormack investigated the relationship between stereoacuity and contrast with a pair of matched TSD monitors by viewing them through a mirror haploscope. The stereo targets were dynamic random-element stereograms with a 50% element density. They found that the correlation threshold at high contrast was independent of contrast, while the correlation threshold at low contrast was inversely proportional to the diagonal square [3].

Although the relationship between contrast and stereoacuity may have been influenced by different test methods and environments (Figure 5), it was somewhat different from that previously outlined in the literature. However, we intended to test the tolerance of stereopsis to variations in luminance and contrast while using different digital display terminals, such as computers, laptops, or smartphones. The luminance and contrast of a display vary from brand to brand and even product to product. If the stereoacuity test results were significantly influenced by luminance and contrast, calibration would be required to ensure a consistent standard. Our experiment determined the opposite trend. Human stereopsis is sufficiently tolerant to variations in luminance and contrast within a relatively large range. The maximum luminance of a display varied between 30 lux and 240 lux in our experiment, which covers commonly used digital displays, and was not a significant factor in this field. Meanwhile, contrast was not an obvious influencing factor, unless it was as low as or below 30%. However, the contrast of monitors would never reach such an extreme, so it is not relevant to the result of the stereoacuity test. Therefore, standardized calibration of luminance and contrast is not necessary for testing stereopsis.

In our research, the difference in stereopsis between random-dot-based and contour-based patterns was not significant under normal test conditions. However, a difference appeared under the relatively low contrast condition, that is, the results for the contour-based pattern were better than those for the random-dot-based pattern. This may be due to different mechanisms between local and global stereopsis. In the extralow contrast condition, the interference for globe stereopsis (tested with random-dot-based pattern) was much more obvious than that for local stereopsis (tested with contour-based pattern). The contour factors may provide useful clues for maintaining stereopsis in low-contrast environments, whereas this was not the case for random-dot patterns. In Fawcett's view, contour-based and random-dot-based patterns had different underlying mechanisms with varying susceptibilities to disruption, and each evaluated a slightly different aspect of the sensory system. The contour-based pattern provided a stimulus to engage fusion mechanisms that were not present in the random-dot-based pattern of the tests [34].

Computer-aided stereoacuity evaluation technology is expected to be more widely accepted in the future owing to its portability, adaptability, and versatility compared with the traditional methods. The limitation of the 3D expression system we established was not that convenient to carry out in the clinic. The requirements for the cooperation of the participants were relatively high. Other 3D expressions, such as shuttle glasses technology or polarized glasses technology, may be limited by the resolution of the display, which is the pixel pitch. It is essential to use a relatively far distance to evaluate stereopsis. The pixel density of computer displays is expected to continue to increase, similar to progressive enhancements of the resolving power of a digital camera sensor. A computer-aided stereoacuity evaluation system, equipped with modern 3D technology, will be applied in many situations, resulting in more sophisticated examinations than that using traditional stereoacuity measurement tools.

## 5. Conclusion

The luminance and contrast of the digital display are not critical factors for stereoacuity under normal circumstances. This implies that a standard monitor with a certain 3D technology can be used to measure the stereoacuity threshold, without calibrating the luminance and contrast.

## Figures and Tables

**Table 1:** Kruskal–Wallis test result of the four groups with different screen luminance.

Contrast (%)	Random-dot-based		Contour-based	
	Chi-square	*P* value	Chi-square	*P* value
95	2.626	0.453	0.283	0.963
90	2.626	0.453	0.283	0.963
85	2.626	0.453	0.283	0.963
80	2.626	0.453	0.283	0.963
75	2.626	0.453	0.283	0.963
70	2.626	0.453	0.283	0.963
65	2.044	0.563	0.283	0.963
60	2.044	0.563	0.283	0.963
55	2.044	0.563	0.283	0.963
50	2.255	0.521	0.260	0.967
45	2.261	0.520	0.260	0.967
40	0.888	0.828	0.327	0.955
35	3.244	0.356	0.715	0.870
30	5.709	0.127	1.345	0.718
25	4.192	0.241	0.677	0.879
20	7.472	0.059	1.909	0.591
15	6.670	0.083	2.828	0.419
10	3.775	0.289	0.533	0.912
5	3.000	0.392	0.365	0.947

**Table 2:** Wilcoxon signed-rank test result between random-dot-based and contour-based measurements in different luminance groups.

Contrast (%)	Group 1 (240 lux)		Group 2 (120 lux)		Group 3 (60 lux)		Group 4 (30 lux)	
	*Z*	*P*	*Z*	*P*	*Z*	*P*	*Z*	*P*
95	−1.155	0.248	−1.000	0.317	−1.508	0.132	−1.748	0.080
90	−1.155	0.248	−1.000	0.317	−1.508	0.132	−1.748	0.080
85	−1.155	0.248	−1.000	0.317	−1.508	0.132	−1.748	0.080
80	−1.155	0.248	−1.000	0.317	−1.508	0.132	−1.748	0.080
75	−1.155	0.248	−1.000	0.317	−1.508	0.132	−1.748	0.080
70	−1.155	0.248	−1.000	0.317	−1.508	0.132	−1.748	0.080
65	−1.155	0.248	−1.633	0.102	−1.508	0.132	−1.748	0.080
60	−1.155	0.248	−1.633	0.102	−1.508	0.132	−1.748	0.080
55	−1.155	0.248	−1.633	0.102	−1.508	0.132	−1.748	0.080
50	−1.155	0.248	−1.633	0.102	−1.604	0.109	−1.780	0.075
45	−1.155	0.248	−1.633	0.102	−1.604	0.109	−1.836	0.066
40	−1.483	0.138	−1.897	0.085	−1.706	0.088	−1.836	0.066
35	−1.589	0.112	−1.897	0.085	−1.897	0.058	−1.906	0.057
30	−1.836	0.066	−1.933	0.053	−2.178	0.029	−3.086	0.002
25	−2.088	0.037	−2.200	0.028	−2.388	0.017	−3.226	0.001
20	−1.995	0.046	−2.390	0.017	−2.688	0.008	−3.138	0.002
15	−2.183	0.029	−2.677	0.007	−3.066	0.002	−3.326	0.001
10	−2.990	0.003	−3.020	0.003	−2.990	0.003	−3.517	<0.001
5	−3.415	0.001	−3.187	0.001	−3.420	0.001	−3.309	0.001

## Data Availability

All the raw data of this article are given in the supplementary tables 1–8.

## Supplementary Materials

Supplementary Materials Test results of stereopsis (Supplementary table).

## References

[1] Legge G. E., Yuanchao G. (1989). Stereopsis and contrast. *Vision Research*.

[2] Halpern D. L., Blake R. R. (1988). How contrast affects stereoacuity. *Perception*.

[3] Cormack L. K., Stevenson S. B., Schor C. M. (1991). Interocular correlation, luminance contrast and cyclopean processing. *Vision Research*.

[4] Leske D. A., Birch E. E., Holmes J. M. (2006). Real depth vs randot stereotests. *American Journal of Ophthalmology*.

[5] Bohr I., Read J. C. A. (2013). Stereoacuity with Frisby and revised FD2 stereo tests. *PLoS One*.

[6] Tejedor J., Ogallar C. (2008). Comparative efficacy of penalization methods in moderate to mild amblyopia. *American Journal of Ophthalmology*.

[7] Maeda M., Sato M., Ohmura T., Miyazaki Y, Wang A. H, Awaya S (1999). Binocular depth-from-motion in infantile and late-onset esotropia patients with poor stereopsis. *Investigative Ophthalmology & Visual Science*.

[8] Reynaud A., Hess R. F. (2018). Interocular correlation sensitivity and its relationship with stereopsis. *Journal of Vision*.

[9] Garnham L., Sloper J. J. (2006). Effect of age on adult stereoacuity as measured by different types of stereotest. *British Journal of Ophthalmology*.

[10] Lee J. Y., Seo J. Y., Baek S. U. (2013). The effects of glasses for anisometropia on stereopsis. *American Journal of Ophthalmology*.

[11] van Doorn L. L. A., Evans B. J. W., Edgar D. F., Fortuin M. F. (2014). Manufacturer changes lead to clinically important differences between two editions of the TNO stereotest. *Ophthalmic and Physiological Optics*.

[12] Zhao L., Stinnett S. S., Prakalapakorn S. G. (2019). Visual acuity assessment and vision screening using a novel smartphone application. *The Journal of Pediatrics*.

[13] Dain S. J., AlMerdef A. (2016). Colorimetric evaluation of iphone apps for colour vision tests based on the ishihara test. *Clinical and Experimental Optometry*.

[14] Hwang A. D., Peli E. (2016). Positive and negative polarity contrast sensitivity measuring app. *Is & T International Symposium on Electronic Imaging*.

[15] Chen J. Y. C., Oden R. V. N., Merritt J. O. (2014). Utility of stereoscopic displays for indirect-vision driving and robot teleoperation. *Ergonomics*.

[16] Khairuddin H. R., Malik A. S., Mumtaz W., Kamel N., Xia L. Analysis of EEG signals regularity in adults during video game play in 2D and 3D.

[17] Oliveira S., Jorge J., González-Méijome J. M. (2012). Dynamic accommodative response to different visual stimuli (2D vs 3D) while watching television and while playing nintendo 3Ds console. *Ophthalmic and Physiological Optics*.

[18] Han S. B., Yang H. K., Kim J., Hong K., Lee B., Hwang J.-M. (2015). New stereoacuity test using a 3-Dimensional display system in children. *PLoS One*.

[19] Jung J.-H., Yeom J., Hong J., Hong K., Min S.-W., Lee B. (2011). Effect of fundamental depth resolution and cardboard effect to perceived depth resolution on multi-view display. *Optics Express*.

[20] Ma D. J., Yang H. K., Hwang J.-M. (2013). Reliability and validity of an automated computerized visual acuity and stereoacuity test in children using an interactive video game. *American Journal of Ophthalmology*.

[21] Westheimer G. (2013). Clinical evaluation of stereopsis. *Vision Research*.

[22] Kim J., Yang H. K., Kim Y., Lee B., Hwang J.-M. (2011). Distance stereotest using a 3-Dimensional monitor for adult subjects. *American Journal of Ophthalmology*.

[23] Wu H., Jin H., Sun Y. (2016). Evaluating stereoacuity with 3D shutter glasses technology. *BMC Ophthalmology*.

[24] Rodríguez-Vallejo M., Ferrando V., Montagud D., Monsoriu J. A., Furlan W. D. (2017). Stereopsis assessment at multiple distances with an IPAD application. *Displays*.

[25] Bonfanti S., Gargantini A., Vitali A. (2015). A mobile application for the stereoacuity test. *Digital Human Modeling. Applications in Health, Safety, Ergonomics and Risk Management: Ergonomics and Health*.

[26] Wu H., Liu S., Wang R. (2018). Stereoacuity measurement using a phoropter combined with two 4K smartphones. *Clinical and Experimental Optometry*.

[27] Sun Y., Wu H., Qiu Y., Yue Z. (2018). Stereoacuity of black-white and red-green patterns in individuals with and without color deficiency. *Journal of Ophthalmology*.

[28] Zhao L., Wu H. (2019). The difference in stereoacuity testing: contour-based and random dot-based graphs at far and near distances. *Annals of Translational Medicine*.

[29] Zhang Y., Meng B., Wu H. (2021). Evaluating the mechanism by which the TNO stereo test overestimates stereo thresholds. *Journal of Ophthalmology*.

[30] Zhao L., Wu H. (2019). Stereoacuity measurement using an auto-stereoscopic smartphone. *Annals of Translational Medicine*.

[31] Yang Y., Wu H. (2019). Screening for stereopsis of children using an autostereoscopic smartphone. *Journal of Ophthalmology*.

[32] Zhao L., Wu H. (2020). The effect of dot size in random-dot stereograms on the results of stereoacuity measurements. *BMC Ophthalmology*.

[33] Li C.-Y., Guo K. (1995). Measurements of geometric illusions, illusory contours and stereo-depth at luminance and colour contrast. *Vision Research*.

[34] Fawcett S. L. (2005). An evaluation of the agreement between contour-based circles and random dot-based near stereoacuity tests. *Journal of American Association for Pediatric Ophthalmology and Strabismus*.

