# Supplementary material for: Effect of Luminance and Contrast Variation on Stereoacuity Measurements Using Smartphone Technology
Source: J Ophthalmol. 2021 Dec 23;2021:5258782. doi: 10.1155/2021/5258782 (PMC8718285; doi:10.1155/2021/5258782)
Supplement: Supplementary Materials — Test results of stereopsis (Supplementary table). [file 5258782.f1.zip › 5258782.f1/Supplementary table 3.docx]

**Table 3. Test results of random-dot-based stereopsis of group 3 (arcsec )**

| ID | Michelson contrast (%) | | | | | | | | | | | | | | | | | | |
| --- | --- | --- | --- | --- | --- | --- | --- | --- | --- | --- | --- | --- | --- | --- | --- | --- | --- | --- | --- |
|  | 95 | 90 | 85 | 80 | 75 | 70 | 65 | 60 | 55 | 50 | 45 | 40 | 35 | 30 | 25 | 20 | 15 | 10 | 5 |
| 1 | 20 | 20 | 20 | 20 | 20 | 20 | 20 | 20 | 20 | 20 | 20 | 20 | 20 | 20 | 20 | 40 | 70 | 80 | - |
| 2 | 30 | 30 | 30 | 30 | 30 | 30 | 30 | 30 | 30 | 30 | 30 | 30 | 30 | 30 | 30 | 30 | 30 | 30 | - |
| 3 | 40 | 40 | 40 | 40 | 40 | 40 | 40 | 40 | 40 | 40 | 40 | 40 | 40 | 40 | 80 | 120 | 210 | 460 | - |
| 4 | 30 | 30 | 30 | 30 | 30 | 30 | 30 | 30 | 30 | 30 | 30 | 30 | 30 | 30 | 30 | 30 | 30 | 40 | - |
| 5 | 30 | 30 | 30 | 30 | 30 | 30 | 30 | 30 | 30 | 30 | 30 | 30 | 30 | 30 | 30 | 30 | 40 | 40 | - |
| 6 | 30 | 30 | 30 | 30 | 30 | 30 | 30 | 30 | 30 | 30 | 30 | 30 | 40 | 40 | 40 | 60 | 50 | 80 | - |
| 7 | 40 | 40 | 40 | 40 | 40 | 40 | 40 | 40 | 40 | 40 | 40 | 50 | 100 | 100 | 100 | 120 | 300 | 640 | - |
| 8 | 40 | 40 | 40 | 40 | 40 | 40 | 40 | 40 | 40 | 50 | 50 | 50 | 50 | 130 | 160 | 180 | 180 | 630 | - |
| 9 | 40 | 40 | 40 | 40 | 40 | 40 | 40 | 40 | 40 | 40 | 40 | 40 | 40 | 40 | 40 | 40 | 50 | - | - |
| 10 | 30 | 30 | 30 | 30 | 30 | 30 | 30 | 30 | 30 | 30 | 30 | 30 | 30 | 30 | 30 | 40 | 40 | 40 | - |
| 11 | 40 | 40 | 40 | 40 | 40 | 40 | 40 | 40 | 40 | 40 | 40 | 40 | 40 | 60 | 60 | 70 | - | - | - |
| 12 | 20 | 20 | 20 | 20 | 20 | 20 | 20 | 20 | 20 | 20 | 20 | 20 | 20 | 20 | 20 | 30 | 30 | 70 | - |
| 13 | 30 | 30 | 30 | 30 | 30 | 30 | 30 | 30 | 30 | 30 | 30 | 30 | 30 | 30 | 30 | 30 | 40 | 40 | - |
| 14 | 30 | 30 | 30 | 30 | 30 | 30 | 30 | 30 | 30 | 30 | 30 | 30 | 30 | 30 | 30 | 30 | 30 | 30 | - |
| 15 | 30 | 30 | 30 | 30 | 30 | 30 | 30 | 30 | 30 | 30 | 30 | 30 | 30 | 30 | 40 | 40 | 70 | 90 | - |
| 16 | 40 | 40 | 40 | 40 | 40 | 40 | 40 | 40 | 40 | 40 | 40 | 40 | 40 | 50 | 50 | 80 | 110 | - | - |
| 17 | 30 | 30 | 30 | 30 | 30 | 30 | 30 | 30 | 30 | 30 | 30 | 30 | 40 | 40 | 50 | 50 | 120 | - | - |
